# Undulatory Swimming Performance Explored With a Biorobotic Fish and Measured by Soft Sensors and Particle Image Velocimetry

**DOI:** 10.3389/frobt.2021.791722

**Published:** 2022-01-03

**Authors:** Fabian Schwab, Fabian Wiesemüller, Claudio Mucignat, Yong-Lae Park, Ivan Lunati, Mirko Kovac, Ardian Jusufi

**Affiliations:** ^1^ Locomotion in Biorobotic and Somatic Systems Group, Max Planck Institute for Intelligent Systems, Stuttgart, Germany; ^2^ Aerial Robotics Lab (ARL), Department of Aeronautics, Imperial College London, London, United Kingdom; ^3^ Materials and Technology Center of Robotics, EMPA, Zürich, Switzerland; ^4^ Laboratory for Multiscale Studies in Building Physics, EMPA, Zürich, Switzerland; ^5^ Soft Robotics and Bionics Lab, Department of Mechanical Engineering, Seoul National University, Seoul, South Korea

**Keywords:** fish, robot, tail, sensor, feedback, flow, PIV, DMD

## Abstract

Due to the difficulty of manipulating muscle activation in live, freely swimming fish, a thorough examination of the body kinematics, propulsive performance, and muscle activity patterns in fish during undulatory swimming motion has not been conducted. We propose to use soft robotic model animals as experimental platforms to address biomechanics questions and acquire understanding into subcarangiform fish swimming behavior. We extend previous research on a bio-inspired soft robotic fish equipped with two pneumatic actuators and soft strain sensors to investigate swimming performance in undulation frequencies between 0.3 and 0.7 Hz and flow rates ranging from 0 to 20 $\frac{cm}{s}$ in a recirculating flow tank. We demonstrate the potential of eutectic gallium–indium (eGaIn) sensors to measure the lateral deflection of a robotic fish in real time, a controller that is able to keep a constant undulatory amplitude in varying flow conditions, as well as using Particle Image Velocimetry (PIV) to characterizing swimming performance across a range of flow speeds and give a qualitative measurement of thrust force exerted by the physical platform without the need of externally attached force sensors. A detailed wake structure was then analyzed with Dynamic Mode Decomposition (DMD) to highlight different wave modes present in the robot’s swimming motion and provide insights into the efficiency of the robotic swimmer. In the future, we anticipate 3D-PIV with DMD serving as a global framework for comparing the performance of diverse bio-inspired swimming robots against a variety of swimming animals.

## 1 Introduction

Bio-inspired and bio-mimetic research have both grown steadily over the last 2 decades and allowed the development of modern, more life-like robots inspired by natural objects. Despite those more sophisticated designs, robots still fall short of the universality and robustness of animal movement and lag behind in important areas such as sensing capabilities and perturbation responses. Geckos run with ease across water (Nirody et al., 2018), crocodiles roll in complex patterns to kill their prey (Fish et al., 2007), and despite continually changing flow conditions and strong locomotor requirements, fish may travel upstream for weeks while fasting (Crossin et al., 2004). Animals outperform robotic platforms and are more resilient than traditional robots in large part because of their compliant structures with integrated sensing capabilities, which enable them to respond to unexpected changes and improve stability through morphological intelligence (Woodward and Sitti, 2018; Siddall et al., 2019; Miriyev and Kovač, 2020; Shield et al., 2021).

Our aim of bio-inspired robotics is threefold: We want to understand the fundamental processes of nature, to develop the ability to mimic parts of it, and eventually to engineer robotic platforms with similar properties. Recent development in soft robotics has attempted to expand on nature’s functionality by creating robots made of materials more akin to those used in living beings (Ijspeert, 2020). The advantages of soft robotics are numerous: their ease of construction, inherent safety, and ability to handle fragile objects or move through unstructured terrains are all promising characteristics. Among the most often used modes of actuation are elastomeric actuators, hydrogels, form memory alloys (SMA), and electroactive polymers (EAP).

One important area of focus for soft robotics is swimming animals and aquatic locomotion, where models of extremely mobile systems can be found (Lauder et al., 2007; Kovač, 2013). Fish are agile swimmers (Colgate and Lynch, 2004), capable of moving in rapidly evolving flow environments and undergoing strenuous locomotion demands such as swimming upstream for weeks while fasting (Crossin et al., 2004). They achieve this high energy efficiency primarily by using the stiffness of their body structure (Lauder et al., 2011) to change the amplitude and frequency of their undulation (McHenry et al., 1995). By matching the frequency of body undulation with the incident flow, they swim efficiently and convert energy from the fluid to the body (Akanyeti et al., 2016; Beal et al., 2006; Liao and Akanyeti, 2017). Caudal fin oscillation is therefore one of the most effective modes of locomotion in terms of transport costs (Ludeke and Iwasaki, 2019; Rayner, 1986), as well as underwater speeds (Block et al., 1992). This results in passive propulsion that propels even dead fish specimens forward (Liao et al., 2003).

The study of these fish locomotion habits culminated in the creation of a number of soft robotics capable of moving in liquids (Struebig et al., 2020; Nguyen and Ho, 2021): Robotic fish mimicking the motion of tuna (Barrett, 1996), even exceeding their hunting speeds (Zhu et al., 2019), robots replicating the rapid “C-start” maneuver seen in carangiform fish (Marchese et al., 2014), or robotic platforms powered acoustically and capable of swimming in three dimensions (Katzschmann et al., 2018, 2016). Additionally, lateral body movements and reflex-base jumping skills have been transferred to robots (Fan et al., 2005; Wright et al., 2019; Kim J. et al., 2020; Zhao et al., 2020; Yang et al., 2021). The mechanisms by which fish use their soft structures, the interaction between active and passive stiffness control, as well as the internal dynamics, are all under-explored and hold significant potential for bio-mimetic technology transfer (Low and Chong, 2010; Low et al., 2010). Although full passive fin systems will advance through water in the same manner as fish, variations in flow velocity or frequency have an impact on thrust output and drag (Jayne and Lauder, 1996; Yun et al., 2011, 2015). To mimic the ability of fish to adjust the amplitude of their swimming body undulations and fully exploit soft surfaces, it is required to control those aspects based on sensory information.

Qualitative hydrodynamic investigations are required to determine how different swimming styles affect swimmers’ thrust performance. The goal of this paper is to enhance studies of living animals by showcasing a simple, robophysical fish platform, enabling the exploration of undulatory locomotion parameters during different swimming and flow conditions, including characteristics not usually seen in swimming animals. Our robophysical fish platform can be used to experimentally investigate questions pertaining to the fundamentals of fish swimming relating Strouhal number to swimming efficiency (Nudds et al., 2014; Eloy, 2012; Floryan et al., 2018) to formulate relevant conservation planning for fish populations in the wild (Link et al., 2017).

Inspired by the rainbow trout, we perform flow experiments with a soft robotic fish platform, consisting of two pneumatically actuated soft actuators and equipped with soft strain sensors. We investigate undulation frequencies ranging from 0 to 1 Hz and flow speeds of 0 to a maximum of 20 $\frac{cm}{s}$ . We first assess the sensor performance of the soft strain sensors, and then explore the controller behavior in the different flow and frequency conditions. We then perform a Particle Imaging Velocimetry (PIV) analysis, measuring the flow angles produced by the undulatory motion, assessing the thrust production of the robot with the wake power model and in the end, we analyze the flow field behind the robotic platform using Dynamic Mode Decomposition (DMD).

With bio-inspired robotics, we can explore new capabilities and hopefully narrow the gap between human-made robots and their natural counterparts. Not only is reverse engineering used to improve robotics, but it also allows biology to test hypotheses that would be impossible to test with live specimens, and hence serves as “model animals” for biomechanics science (Siddall et al., 2021; Nyakatura et al., 2019). The present study provides insights into the control strategies adopted from the rainbow trout and the resulted thrust generated.

## 2 Methods

**FIGURE 1:**
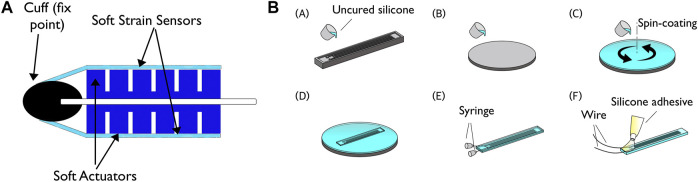
(a) Schematic of soft robotic fish platform (b) Soft sensor fabrication process: **(A)** Uncured silicone rubber is poured into the 3D printed mold. **(B)** To fabricate the unpatterned second layer, uncured liquid silicone is coated on a flat substrate. **(C)** To bond the layers, the flat layer is spin-coated with uncured silicone and partially cured at room temperature. **(D)** The layer with the micro-channel-pattern is gently placed on the flat layer, and the combined structure is cured again. **(E)** After bonding, eGaIn is injected into the micro-channels using two syringes. **(F)** Two wires are connected and securely attached with a silicone adhesive. Image reproduced from (Lin et al., 2021).

The proposed soft-robotic system is composed of a robotic fish, consisting of two soft pneumatic actuators that are attached to a flexible panel with stiffness comparable to that of a fish body and equipped with integrated eutectic gallium–indium (eGaIn) sensors (Figure 1A). A PID controller was used to control the undulation movement and conduct velocity and frequency sweeps in a recirculating flow tank.

### 2.1 Soft Robotic Fish Platform With eGaIn Sensors

**FIGURE 2:**
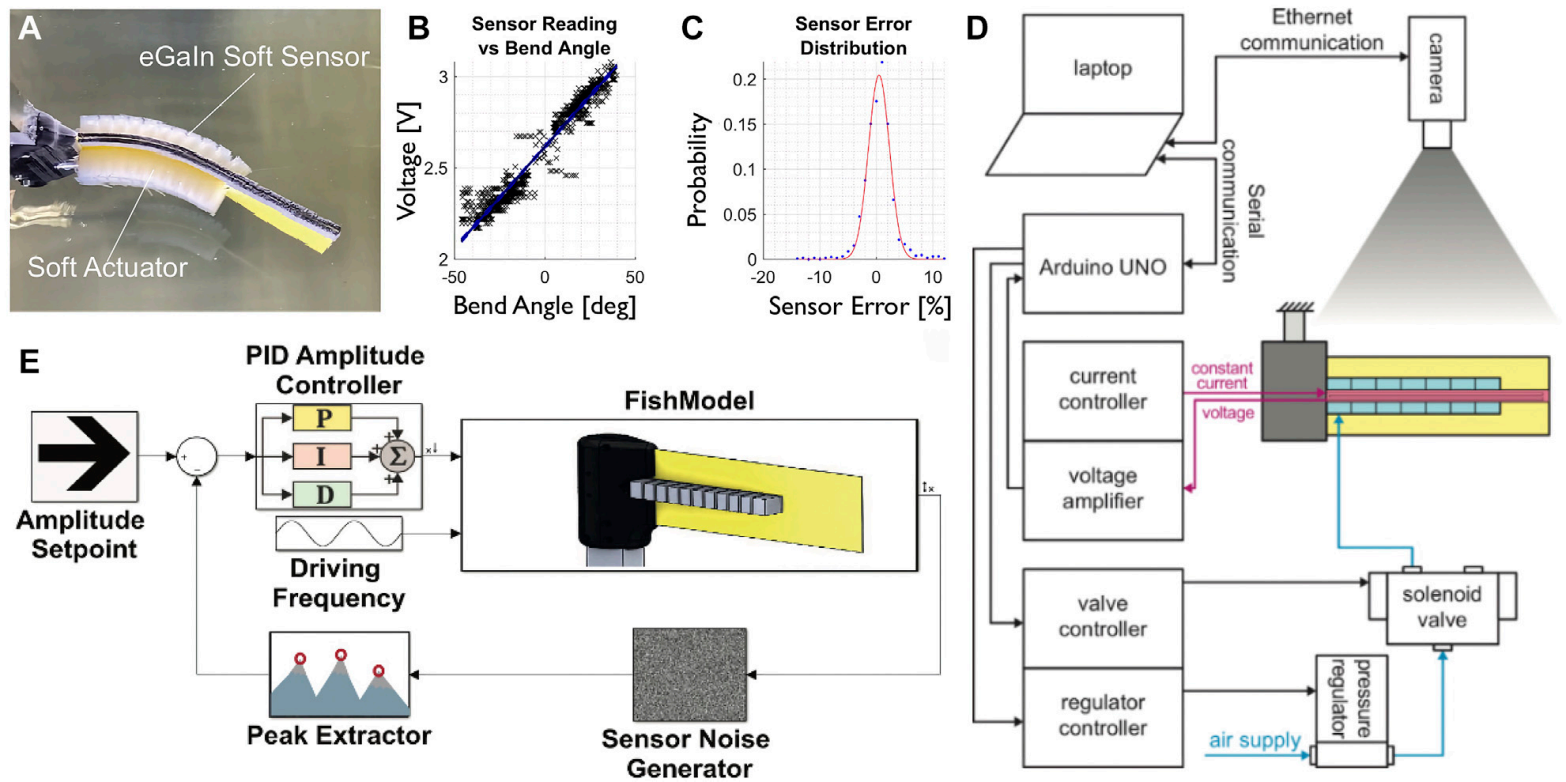
Overview of the robot and its performance. **(A)** Top view of soft robotic fish with soft actuator and sensor (Lin et al., 2021). **(B)** Sensor linearity, sampled at 100 Hz over 1s of undulation at 1 Hz (Lin et al., 2021). **(C)** Sensor error histogram, with a fitted Gaussian distribution (Lin et al., 2021). **(D)** Overview of all the components of the setup to control the soft robotic fish. **(E)** Illustration of the controller implemented to adapt to external disturbances. All images reproduced and modified from (Lin et al., 2021).

A simple, fish-like geometry was constructed representing the spine and fin of the animal, with a core plastic sheet (Plastic Shim Stock™  0.5 mm, Artus). A silicone fast-PneuNet actuator molded with uncured elastomer (Dragon Skin™ 20, Smooth-On) (Mosadegh et al., 2014), 10 cm in length and 2 cm in height, was attached with a cured silicone sealant (ELASTOSIL™ E43, Wacker) to both sides of the sheet, representing the muscles of the fish for lateral undulation motion. The segmented and chambered soft actuator allows for a smooth bending pattern, similar to the undulation motion of fish (Figure 1A; Figure 2A).

The fabrication process for the soft strain sensors is shown in Figure 1B, based on the work from (Park et al., 2010). For the first layer with the microchannel pattern, uncured silicone rubber (EcoFlex™ 0030, elastic modulus E = 125 kPa) is poured into a 3D printed (Objet30™  Objet Geometries Ltd.) mold. For the second layer, uncured liquid silicone is coated on a flat substrate with a thickness of 1 mm. Both layers are than cured for an hour at 60°C in a convection oven. The flat layer is then spin-coated (1,000 rpm for 60 s) with uncured silicone and partially cured at room temperature for 10 min. Then, the layer with the microchannel pattern is gently placed on the flat layer, and the combined structure is cured again.

After bonding, conductive liquid metal eutectic Gallium-Indium (eGaIn, Sigma-Aldrich) is injected into the microchannels using two syringes, one for injection and the other to release the air captured in the channels during fabrication. Two wires are connected to both ends of the microchannel via the syringe holes. Finally, a silicone glue (Sil-Poxy, SmoothOn™) is applied and cured around the holes into which the wires are inserted, firmly holding the wires and sealing the holes.

Changes in the cross-sectional region of the eGaIn-filled channels result in changes in electrical resistance, which can be used to measure the bending amplitude of the soft robot. The sensors have low temperature sensitivity (Vogt et al., 2013) and the liquid’s incompressibility makes them ideal for water experiments with change of ambient pressure (Hellebrekers et al., 2018; Schwab et al., 2021). For calibration testing, the sensor was bent in the undulation range of −50 to 50°, and during this angle sweep the voltage was sampled. In comparison to the ground truth kinematics obtained from videography, the sensor response was linear (R2 = 0.952, Figure 2B), with a relative error well described by Gaussian noise with a standard deviation of 0.4% (Figure 2C).

In this work, hook-and-loop fasteners instead of embedded fabric like in (Mengüç et al., 2014) were used, for quick adjustment and replacement of the sensors onto the soft actuators.

### 2.2 Model and Controller Design

To characterize swimming robots, various models for undulatory locomotion have been proposed (Salumäe et al., 2012; Bliss et al., 2013). In this paper, a data-driven, lumped parameter model was used, which is a popular method when modeling compliant robots (Sadler and Sandor, 1973; Wang and Huston, 1994; Nishikawa et al., 2015). The soft silicone robot was treated as a chain of rigid elements, which are connected by hinges with constant stiffness and damiping coefficients. The parameter of the hinges were then optimized with genetic algorithm using experimental ground-truth kinematics (Lin et al., 2021). Compared to finite element simulations (Allard et al., 2007) or machine learning tools (Gillespie et al., 2018), this data-driven approach is computationally less demanding while still managing to capture the behavior of the soft robotic fish (Lin et al., 2021).

The model uses the pressure as input and takes the co-contraction effects as well as hydrodynamic forces into account, successfully predicting the angle and position of the soft robotic fish and simulating the ground-truth behavior. This enables a faster testing of a controller, without the need of constant experiments for verification. In the future, the model will hopefully accelerate more sophisticated control designs and guide the further development of soft swimming robots.

The amplitude control system is designed in Simulink (MATLAB™ R2020b) and a flow chart is shown in Figure 2E. A proportional-integral-derivative (PID) controller is used for pressure adjustments in response to the measured curvature. Fish can be observed to adjust their undulation frequency to different flow conditions, while their amplitude remains constant (Tytell and Lauder, 2004). The goal of the controller is therefore to maintain a specific amplitude across a range of undulation frequencies and flow speeds, by controlling the pressure. The verification of the controller performance was previously done in a static tank (Lin et al., 2021) (Figure 2A) and we now evaluate the output of such a controller in a flow tank with varying flow rates.

Figure 2D shows the components for the controller and the experimental setup. Through a digital pressure regulator (ITV0050-3BS, SMC), 2.5 bar compressed air is supplied to the system. The PneuNet actuators are controlled by two directional solenoid valves (SYJ7320-5LOU-01F-Q, SMC) respectively. A micro-controller (ATmega328 on Arduino Uno, Arduino) sends the control signals to the valves as well as to the pressure regulator. The robotic platform is mounted on a 150 mm long, 20 × 30 mm rectangular aluminum section with a 2 mm wall thickness. Currently, the controller’s response is limited by the use of peak amplitude, which suggests that the control input is changed only twice per oscillation cycle.

### 2.3 Experimental Setup

**FIGURE 3:**
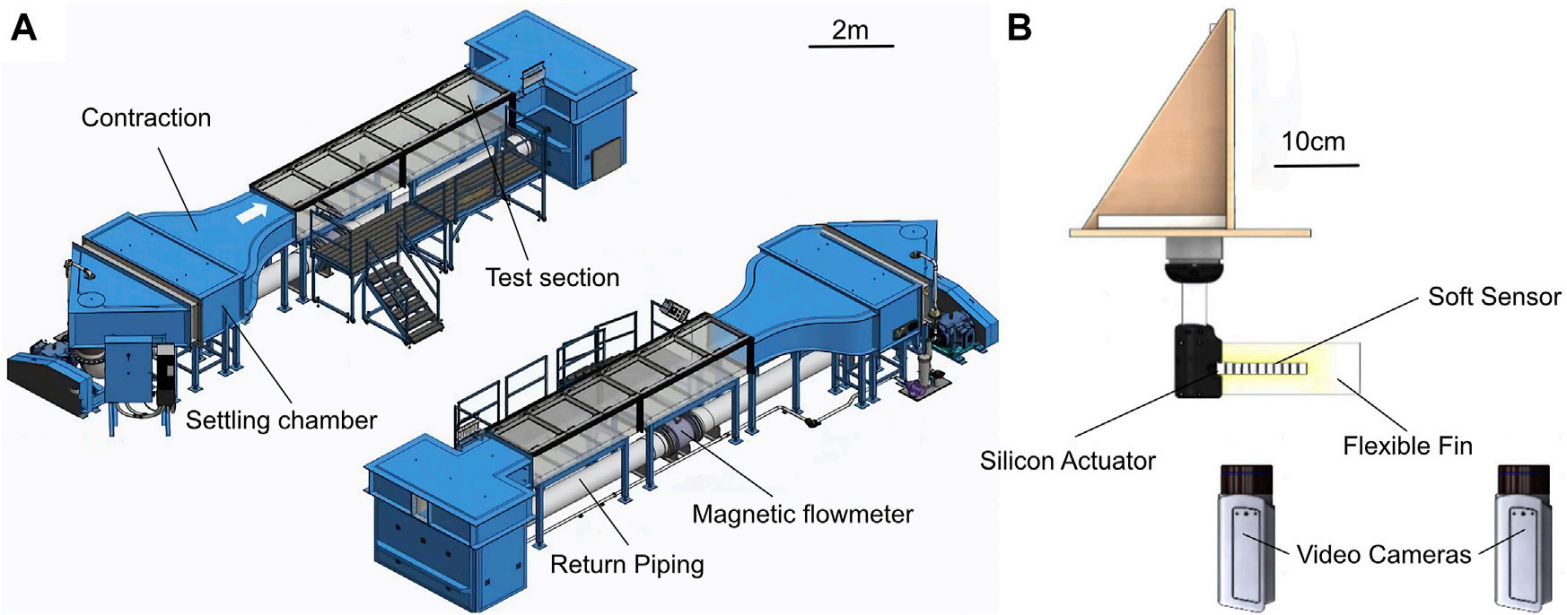
Experimental setup at the Flow Tunnel at Empa Zürich for the PIV experiments. **(A)** General setup of the flow tunnel. **(B)** The fishrobot was placed in the water flow tank, and different flow speeds as well as undulation frequencies have been tested.

Particle Image Velocimetry (PIV) (Willert and Gharib, 1991) is an established method used to study flow phenomena (Sfakiotakis et al., 1999) and is well adapted for investigating the physics surrounding the soft robotic fish platform discussed in this paper. Experiments were conducted at Swiss Federal Laboratories for Material Science large scale water flume (Engineering Laboratory Design, Inc.). This facility has a cross section of 0.6 × 1 m^2^ and a fully transparent, 6 m long test section (Figure 3A). The system is driven by a variable speed pump, allowing precise control of the water bulk speed from 0.02 m/s to 1.5 m/s. Controlled velocity profiles at the inlet and outlet of the test section are ensured by a 6:1 inlet contraction and outlet diffuser with guide vanes.

The tail motion and velocity fields were captured using a PIV system that consists of a pulsed Nd:YAG laser, double cavity, with 100 mJ per pulse at 532 nm and a maximum repetition rate of 100 Hz and two 5.5 Mpx SCMOS cameras. The laser beam was guided through a laser arm to the sheet optics, allowing to illuminate a cross section of the robotic fish and the PIV tracers on a horizontal plane at 1/3 the foil height from the bottom. The PIV cameras were positioned underneath the water flume floor and calibrated simultaneously with a 3D calibration target. The cameras were run in double frame mode at 24 fps and equipped with 50 mm focal-length lenses achieving a spatial resolution of 10 px/mm. The combined field of view of both cameras covered an area of 400, ×, 200 mm, aligned with the flow direction. The field of view of Camera 1 was centered on the robotic fish, whereas Camera 2 was used to retrieve the velocity field at the trailing edge of the flexible foil. The origin of the coordinate system was placed at the leading edge of the robotic fish.

The position of the tail of the robotic fish was retrieved by segmentation and thresholding of the recorded images with an algorithm developed in MATLAB. Thanks to short pulsed laser illumination (5 ns pulse duration) the images showed little or no blurring, allowing us to reconstruct the position of the tail with an accuracy of ± 1 mm.

For the PIV measurements, spherical, glass seeding particles of 10 microns were used, while the laser sheet thickness was about 2 mm, in view of the strong 3D structure of velocity field. The images were stitched side-by-side and processed with a multi-grid approach having final interrogation window size of 32 × 32 Px and 50% overlap, yielding to a physical resolution of 0.625 velocity vectors/mm. For each trial, 1,000 images have been recorded to enable the calculation of the mean and rms velocity field, as well as Dynamic Mode Decomposition (DMD) (Schmid, 2010) of the velocity field.

### 2.4 Test Parameters

The closed loop controller of the soft robotic fish has been tested at different flow conditions. First the water flow speed has been adjusted step-wise from 0 $\frac{cm}{s}$ to 19.4 $\frac{cm}{s}$ , while the actuation frequency was kept constant at 0.55 Hz. Afterwards, the actuation frequency has be varied step-wise from 0.3 to 0.7 Hz, while the water flow speed has been left constant at 5.5 $\frac{cm}{s}$ . The exact test parameters of the water flow speed sweep and of the frequency sweep are given in Table 1 and 2, respectively. Before varying the flow speed and frequency a reference trial (No. 1 and 7) at 0 $\frac{cm}{s}$ and a frequency of 0.55 Hz have been performed.

**TABLE 1:** Flow speed sweep.

Trial No	Flow [ $\frac{cm}{s}$ ]	Freq. [Hz]
1	0	0.55
2	5.3	0.55
3	8.8	0.55
4	12.3	0.55
5	15.8	0.55
6	19.4	0.55

**TABLE 2:** Frequency sweep.

Trial No	Flow [ $\frac{cm}{s}$ ]	Freq. [Hz]
7	0	0.55
8	5.5	0.3
9	5.5	0.4
10	5.5	0.5
11	5.5	0.6
12	5.5	0.7

## 3 Results and Discussion

The relationship of the amplitude, frequency, and water flow speed is investigated by examining the strain sensor reading, the tail location derived from the film, and the flow field visualized by PIV. To analyze a wide range of the characteristics of the soft robot, a water flow speed sweep between 5 $\frac{cm}{\sec}$ and 20 $\frac{cm}{\sec}$ as well as cyclic undulation frequencies of the soft fish platform were tested ranging from 0.3 to 0.7 Hz.

### 3.1 Sensor Performance

**FIGURE 4:**
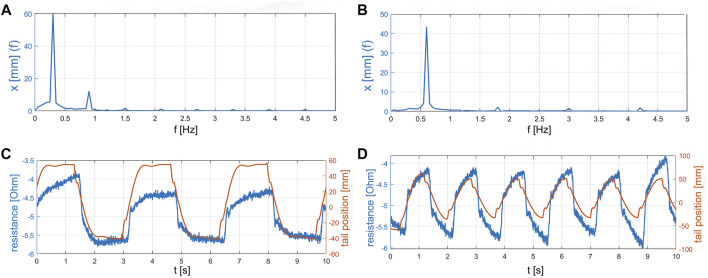
Comparison of sensor results and video analysis of the tail position during trial 8 and 11. **(A)** The Fast Fourier transform spectrum of closed loop trial 08 at 0.3 Hz undulation input frequency. **(B)** The Fast Fourier transform spectrum of closed loop trial 11 at 0.6 Hz undulation input frequency. **(C)** The measured resistance over time compared to the tail position extracted from the video of trial 08. **(D)** The measured resistance over time compared to the tail position extracted from the video of trial 11.

Figure 4 shows the sensor reading performance for two exemplary trials: trial 8 was performed at a undulation frequency of 0.3 Hz and a constant flow velocity of 5 $\frac{cm}{\sec}$ (Figures 4A,C), while trial 11 (Figures 4B,D) was conducted at a frequency of 0.7 Hz. By performing a Discrete Fourier transform of the measurements of the undulatory soft robot, we observed rather high second harmonics in at 0.3 Hz (Figure 4A) compared to 0.6 Hz (Figure 4B). Those second harmonics are hypothesized to be introduced by unwanted structural interactions between the robot’s center plastic sheet and the actuators.

### 3.2 Flow Field Measurements

**FIGURE 5:**
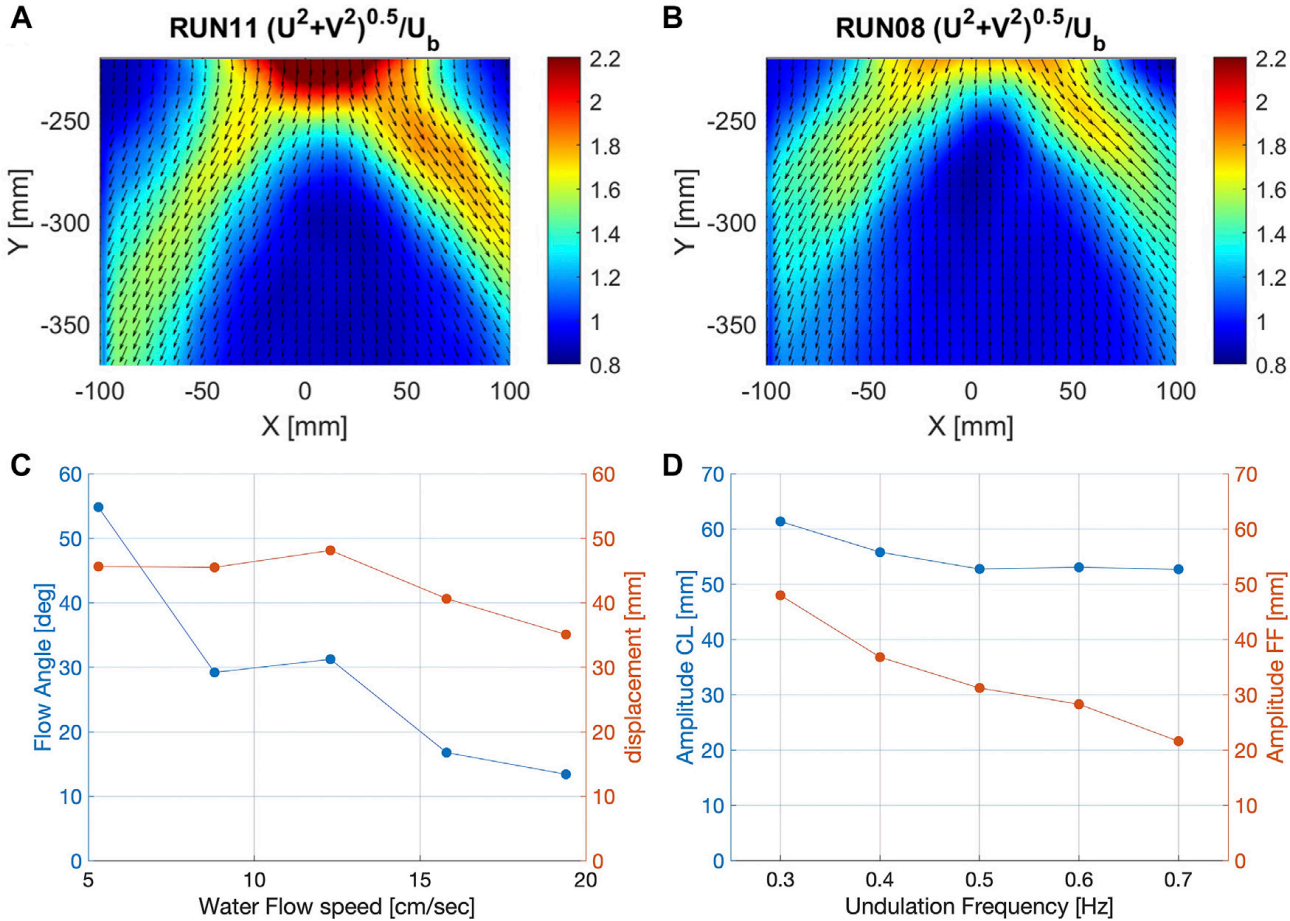
Comparison of the average flow fields at different undulation frequencies, indicating two water jets deflected at changing angles. **(A)** Average flow field during closed loop control and 0.3 Hz undulation frequency (narrow angle). **(B)** Average flow field during closed loop control and 0.6 Hz undulation frequency (wide angle). **(C)** Water flow angle measured at x = 233 mm and tail displacement depending on the water flow. **(D)** Comparison of Amplitude depending on the undulation frequency for open loop (FF) and closed loop (CL).

We analyzed the average flow speed around the swimming fish by PIV experiments. In the time averaged flow field, a clear upside V-shape pattern can be observed (Figures 5A,B), with two distinct jets originating from the tail motion. The angle enclosed by the jets varies in the different trials.

By plotting the angle enclosed by the jets as a function of the different flow parameters, we can illustrate how they affect the time-average features of the flow field associated to the tail motion. Figure 5C depicts the flow angle and displacement as a function of the water flow speed in the recirculating flow tank. The closed loop control is able to adjust the power input to the water flow speed, maintaining a rather constant displacement up to a water flow speed of 15 $\frac{cm}{s}$ .

Figure 5D compares the performance of the robotic platform in feed forward and closed loop control over a range of frequencies. We can observe that controller keeps the amplitude of the soft fish approximately constant while in feed forward control, the amplitude drops significantly with increasing frequency. This illustrates the potential of the controller to mimic the swimming behavior of fish, adapting the parameters to keep a constant tail-tip amplitude in changing environments.

### 3.3 Wake Power

Although the actual flow field exhibits a 3D structure, an approximate estimate of the wake power (the net energy flux) can be obtained from the 2D PIV measurements. Previous experimental work using flapping foils (Triantafyllou et al., 2000) and swimming live fish (Fish and Lauder, 2005) demonstrates that the Strouhal number, a fundamental dimensionless parameter, dominates the hydrodynamic performance of fish locomotion, because it correlates with vortex shedding dynamics (Eloy, 2012; van Leeuwen et al., 2015).

$$S_{t}=\frac{fA_{tail}}{v_{swim}},$$

where *v*_*swim*_ is the mean swimming speed during undulatory movements, *A*_*tail*_ is the peak-to-peak amplitude of the tail tip, and *f* the frequency.

Studies suggest, that the optimal number is in general optimal between 0.1 and 0.55 St, (0.25–0.35 for carangiform swimmers and 0.4–0.5 for anguilliform swimmers) (Nudds et al., 2014; Taylor et al., 2003; Floryan et al., 2018).

Assuming a steady-state, uniform velocity profile upstream and a 2D flow field of thickness equal to the height of the robot, the wake power was estimated by integrating the kinetic energy measured by PIV analysis (TytellSchultz and Webb, 2002; 2007). We computed the kinetic energy flux under 2D assumption from the PIV averaged flow fields on a control volume surrounding the swimmer. In this case the upstream input can be expressed as $\frac{1}{2}\rho hwU^{3}$ , where *U* is the flow speed, *h* the height of the robot, and *w* the width of the wake. Therefore, the net wake power can den be expressed by

$$P_{\text{wake}}^{\prime }=\frac{1}{2}\rho U\int \left[{\left(U+u\right)}^{2}+v^{2}+w^{2}-U^{2}\right]dS$$

where *u*, *v* and *w* are the axial, lateral and vertical fluid velocities produced by the robot, respectively. The power coefficient *C*_*p*_ is obtained by normalizing the wake power by $\frac{1}{2}\rho 2\;hLU^{3}$ , where *L* is the length of the swimmer ((Schultz and Webb, 2002; Tytell and Lauder, 2004; Krueger, 2006)).

**FIGURE 6:**
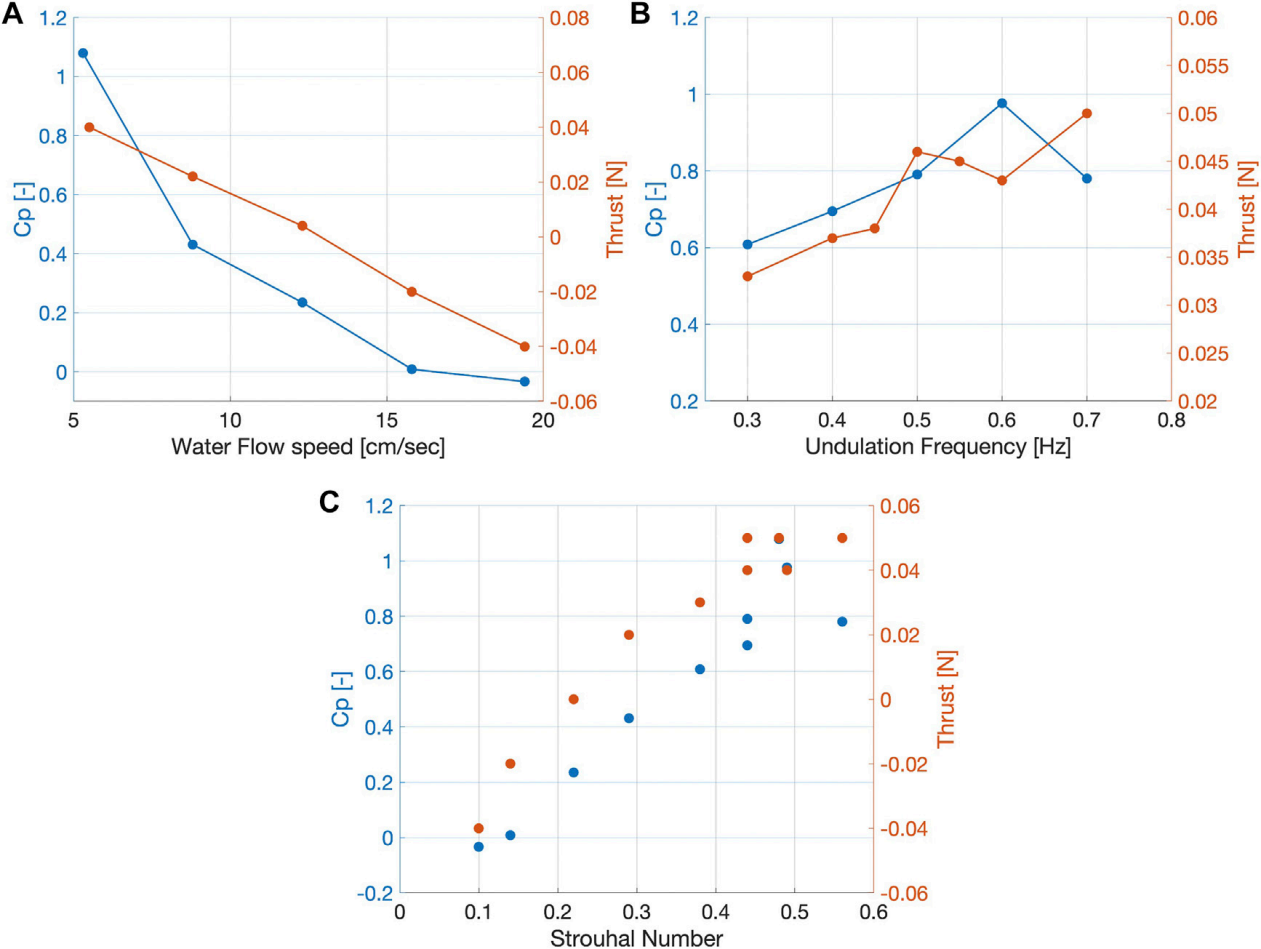
Wake power coefficient and Thrust in relation to the water flow speed and to the undulation frequency (Data for thrust from (Jusufi et al., 2017). **(A)** *C*_*p*_ and thrust decreasing with increasing water flow speed. **(B)** *C*_*p*_ and thrust increase with increasing undulation frequency. *C*_*p*_ reaches maximum at 0.6 Hz, thrust at 0.7 Hz (at flow speed of 5.3 $\frac{cm}{s}$ ). **(C)** A value range similiar to swimming fish can be observed for the Strouhal Number, which measures the efficiency of the swimmer. Values for C_P and Thrust agree, with a local maximum at 4.5 St.

In the present case we measured a *C*_*p*_ close to zero at a water speed of roughly 15 $\frac{cm}{s}$ , which would indicate an estimate of the maximum robotic fish swimming speed. At 5.3 $\frac{cm}{s}$ , we measured an optimal actuation frequency of 0.6 Hz. In Figure 6, we additionally plotted the results from open-loop experiments with a force sensor attached to the robotic fish platform (Jusufi et al., 2017). We can observe that the results from the PIV experiments agree qualitatively with the results from previous experiments. The slight differences might be explained, besides the limitations of this approach, by the new controller implemented in this study, which leads to a slightly different optimal frequency (0.6 Hz compared to 0.8). Furthermore, the wake power model estimates an higher self propelled speed as the *C*_*p*_ equal zero at roughly 15 $\frac{cm}{s}$ , while the previously measured thrust equals zero at 13 $\frac{cm}{s}$ .

In Figure 6C, it can be observed that as the *St* number increases, the thrust as well as the *C*_*p*_ gradually increase from negative to positive values.

It should be stressed that this analysis of 2D data is based on simplistic assumptions and provides only a rough estimate of the wake power and the power coefficient, providing just an indication of the expected swimming speed and the optimal actuation frequency. More accurate estimates require measuring the full 3D flow field. Nevertheless, these results show that a thrust assessment is possible without attaching sensors. We envision to use this method with a range of swimmers like e.g., eels, bluegills, or trouts.

### 3.4 Dynamic Mode Decomposition Analysis of the Velocity Field

Dynamic Mode Decomposition (DMD) is an emerging purely data-driven technique that provides linearly reduced order models for high-dimensional, complex systems (SchmidRowley et al., 2009; 2010; Tu et al., 2014; Kutz et al., 2016). Coupled spatio-temporal coherent patterns or modes can be extracted from the observed data. DMD has applications in fluid dynamics, neuroscience, robotics, or disease modeling.

**FIGURE 7:**
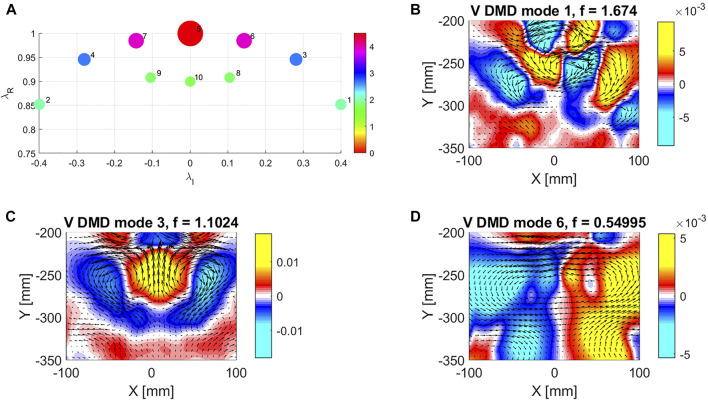
Dynamic mode decomposition (DMD) of flow field behind robotic fish. **(A)** Real and Imaginary parts of the 10 DMD modes used for the decomposition. Circles are color coded based on the modes’ amplitude. **(B–D)** Contour map of the vertical velocity component and velocity vectors for DMD mode 1, mode 3 and mode 5.

As the swimming locomotion is periodic, DMD analysis can be useful to extract the dominant space-time modes of the flow field. As an example, Figure 7A shows the real and imaginary parts of the eigenvalues associated to each spatial mode for an undulation frequency of 0.55 Hz and a free stream velocity of 9.3 $\frac{cm}{s}$ . Besides the zero-frequency mode 7, which represents the average velocity field, we can identify modes 1, 3, and 5 as well as theirs conjugates 2, 4, and 6 that dominate the instantaneous flow field as in view of their amplitude.

Mode 1 (Figure 7B) has a frequency equal to 1.7 Hz, roughly three times the undulation frequency. This mode is associated to the presence of a vortex centered at x = 0, y = − 260 mm and shed by the tail motion at a frequency equal to three times the swimmer undulation frequency. Mode 3 (Figure 7C) has a frequency equal to 1.1 Hz or twice the undulation frequency. As the vertical velocity contour map is symmetric, we expect this mode to be associated to an instantaneous thrust that oscillates periodically between negative and positive values, influencing the instantaneous propulsion. Mode 5 (Figure 7D) has a frequency equal to the undulation frequency (0.55 Hz), and it is directly related to the oscillatory motion of the tail. As the vertical velocity contour map is anti symmetric, we do not expect this mode to be directly linked to a positive or negative trust along the stream wise direction. On the contrary, the lateral component of the thrust vector should be non-zero. We observed these modes also in the other trials, performed with different operating parameters.

## 4 Conclusion

This article extends the pneumatically-actuated system from (Jusufi et al., 2017) with strain sensors described in (Park et al., 2010, 2012) to create a fully soft pneumatic platform. The real-time data from the soft sensors are in agreement with the observed motion analysis and show robust, noise-tolerant real-time curvature measurements. The platform’s swimming performance was tested in a flow tank and the robotic fish is able to maintain a constant amplitude of the tail motion over a wider range of different water flow speeds and undulation frequencies compared to the feed forward approach used in (Jusufi et al., 2017). controller was able to keep a constant tip–tip deflection.

We further show that the Wake Power Model is able to qualitatively measure thrust exerted by the fish platform without the need of attaching external force sensors.

Furthermore, DMD analysis was explored to illustrate the relevant modes of the flow field, showing potential to understand the swimming dynamics of bio-mimetic robots. Here, we focused on the design and characterization of a single soft actuator on each side, although we envisage integrating multiple soft actuators sequentially. In the future, we plan to couple 3D velocity measurements, pressure field/thrust reconstruction and DMD to investigate how the geometry, the mechanical properties, and the control strategy of the swimmer affect swimming performance. We also envision to enhance the features of the soft strain sensors (e.g., the sensitivity durability or flexibility) and use the multi-sensing techniques presented in (Kim T. et al., 2020). A future goal is to further integrate the sensor in soft actuators and improve the interface between biocompatible materials and highly stretchable sensors to provide excellent adhesion. This would make it possible to investigate how the modulation of body stiffness may reduce internal bending resistance, and matching the undulation frequency to specific flow speeds is used to improve swimming speed as well as energy storage (Akanyeti et al., 2016).

With robophysics we can explore new capabilities with a goal to narrow, if not close, the capability gap between human-made robots and their natural counterparts.

## Data Availability

The data presented in the study are deposited in the repository: https://github.com/fabnschwab/Data_PIV2021 repository, accession number bbf72b7.
